# The Nexus of Stemness, Telomere Stability, and Metabolic Reprogramming in Glioblastoma: Foundations of Tumor Persistence and Targets for Intervention

**DOI:** 10.1002/mco2.70693

**Published:** 2026-03-30

**Authors:** Ji‐Yong Sung, Kihwan Hwang

**Affiliations:** ^1^ Department of Neurosurgery Seoul National University Bundang Hospital, Seoul National University, College of Medicine Seongnam Republic of Korea

**Keywords:** alternative lengthening of telomeres (ALT), glioblastoma, glioma stem‐like cells (GSCs), metabolic reprogramming, telomere maintenance mechanisms (TMMs), therapeutic resistance, tumor immune microenvironment

## Abstract

Glioblastoma (GBM) is a highly aggressive, therapy‐resistant brain tumor with inevitable recurrence despite maximal multimodal treatment. Increasing evidence suggests that this intractability arises from coordinated cellular programs rather than a single dominant pathway. Central to these programs are glioma stem‐like cells (GSCs), which sustain self‐renewal, phenotypic plasticity, and resistance to genotoxic and metabolic stress, and yet the molecular basis of their long‐term tumor‐propagating capacity remains incompletely understood.

Here, we synthesize recent advances to propose an integrated conceptual framework—the Triadic Nexus—in which GSC stemness, telomere maintenance mechanisms, and metabolic reprogramming function as a self‐reinforcing regulatory system. We review how telomerase reactivation versus alternative lengthening of telomeres (ALT) differentially shape genomic stability, immune signaling, and metabolic states and how metabolic plasticity feeds back to regulate stemness and telomere‐associated stress responses. Drawing on single‐cell, spatial, and multi‐omics studies, we highlight how these interdependent axes collectively sustain therapy resistance and tumor recurrence.

Finally, we discuss the translational implications of the Triadic Nexus, emphasizing rational combinatorial therapeutic strategies and biomarker‐guided patient stratification based on telomere and metabolic signatures. By unifying stemness, telomere biology, and metabolism into a mechanistically testable model, this review provides a systems‐level framework for understanding GBM persistence and guiding next‐generation therapeutic interventions.

## Graphical abstract

Integrated schematic depicting the interplay between glioma stem‐like cells, telomere maintenance mechanisms, and metabolic reprogramming in shaping immune suppression and therapeutic resistance in glioblastoma

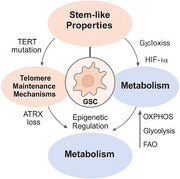

## 1 Introduction

Despite maximal multimodal therapy, glioblastoma (GBM) remains fundamentally incurable, with inevitable recurrence driven by intrinsic cellular plasticity and therapy‐resistant subpopulations. GBM is the most aggressive primary brain tumor in adults, characterized by rapid progression, extensive intratumoral heterogeneity, and dismal clinical outcomes despite advances in surgical resection, radiotherapy, and chemotherapy. From an epidemiological perspective, GBM accounts for approximately 45%–50% of malignant primary brain tumors, with an estimated annual incidence of three to four cases per 100,000 individuals worldwide [1].

Median survival remains approximately 14–15 months [2], underscoring that current treatment paradigms fail not simply because of incomplete tumor removal, but because GBM possesses deeply embedded biological programs that enable persistence under therapeutic pressure.

A growing consensus supports the glioma stem‐like cell (GSC) [3] hypothesis, positioning GSCs as the cellular bedrock of tumor recurrence and therapeutic failure. GSCs represent a functionally distinct subpopulation endowed with self‐renewal capacity, phenotypic plasticity, and intrinsic resistance to genotoxic, metabolic, and oxidative stress. These properties allow GSCs to survive cytotoxic therapies and subsequently regenerate the heterogeneous tumor bulk. Importantly, GSCs are not a static population; instead, they dynamically adapt to fluctuating microenvironmental conditions, suggesting that recurrence is driven by adaptive cellular programs rather than fixed genetic alterations alone.

Historically, the biological processes underlying GBM persistence have been investigated through compartmentalized frameworks, in which stemness is viewed primarily as a transcriptional hierarchy, telomere maintenance as a mechanism conferring replicative immortality, and metabolic reprogramming as a supportive bioenergetic adaptation. While each of these hallmarks is individually essential, increasing evidence indicates that none operates in isolation. Telomere maintenance mechanisms (TMMs) influence cellular metabolism and immune signaling [4], metabolic flux regulates epigenetic and transcriptional programs that sustain stemness, and stemness‐associated transcription factors reciprocally shape both telomere dynamics and metabolic pathway selection. These observations highlight the limitations of reductionist models and point toward a systems‐level organization that underlies GBM persistence.

Importantly, the biological basis of GBM persistence cannot be adequately explained by examining stemness, telomere maintenance, or metabolism in isolation. Rather, accumulating evidence indicates that these processes are functionally interdependent and collectively sustain therapy resistance and tumor recurrence [5, 6]. In this review, we propose an integrated framework—the Triadic Nexus—in which GSC stemness [7, 8], TMMs, and metabolic reprogramming form a self‐reinforcing regulatory system. This conceptual framework provides a coherent biological logic that connects molecular, metabolic, and cellular observations into a unified model of tumor persistence.

Accordingly, this review first summarizes the molecular and cellular foundations of GSC biology, TMMs, and metabolic reprogramming as core pillars of GBM malignancy. We then integrate these elements within the Triadic Nexus framework, systematically dissecting how bidirectional and feed‐forward interactions among the three axes converge to form a self‐reinforcing, therapy‐resistant loop [9]. Finally, we discuss the translational implications of this integrated model, highlighting rational combinatorial therapeutic strategies, biomarker‐guided patient stratification, and emerging technologies‐such as single‐cell and spatial multi‐omics‐that may enable clinical exploitation of Nexus‐derived vulnerabilities.

## 2 The Pillars of GBM Malignancy

### 2.1 GSCs: The Architects of Recurrence

Glioma stem‐like cells (GSCs) constitute a rare but functionally dominant subpopulation within GBM, endowed with the capacity for long‐term self‐Johnsonrenewal, multilineage differentiation, and sustained tumor propagation. Originally identified through their tumor‐initiating potential in orthotopic xenograft models [10], GSCs are now recognized as central drivers of therapeutic resistance and inevitable tumor recurrence. Unlike the rapidly proliferating bulk tumor cells that are effectively debulked by surgery, radiotherapy, and chemotherapy, GSCs persist under cytotoxic stress and reconstitute the heterogeneous tumor architecture upon treatment cessation [11, 12].

A defining feature of GSCs is their intrinsic resistance to diverse therapeutic insults, including DNA damage, oxidative stress, and metabolic perturbation. This resistance is mediated by multiple mechanisms, such as enhanced DNA damage repair capacity, activation of anti‐apoptotic signaling pathways, efficient reactive oxygen species (ROS) detoxification, and quiescent or slow‐cycling cell states. Collectively, these traits allow GSCs to survive conditions that are lethal to non‐stem tumor populations, positioning them as the cellular reservoir from which recurrence emerges [13].

Crucially, GSCs should not be viewed as a rigid or hierarchically fixed population [14, 15]. Accumulating evidence from lineage tracing, single‐cell transcriptomics, and spatial profiling indicates that stemness in GBM is a dynamic and reversible cellular state. Differentiated tumor cells can reacquire stem‐like features in response to microenvironmental cues such as hypoxia, nutrient deprivation, inflammatory signaling, or therapeutic stress. This plasticity challenges classical unidirectional cancer stem cell models and suggests that stemness represents an adaptive phenotype rather than a permanently encoded cell identity.

At the molecular level, GSC identity is orchestrated by a core transcriptional network involving stemness‐associated regulators such as SOX2, OLIG2, NANOG, and POU3F2 [16, 17]. These factors sustain self‐renewal programs while suppressing terminal differentiation. However, transcriptional control alone is insufficient to explain the durability and adaptability of GSCs. Increasing evidence indicates that stemness programs are tightly coupled to cellular metabolism, redox homeostasis, and genome maintenance pathways, allowing GSCs to continuously recalibrate their state in response to environmental pressure.

Importantly, GSCs occupy specialized niches within the tumor microenvironment, including perivascular and hypoxic regions [18], which actively reinforce stem‐like phenotypes [19]. Endothelial‐derived signals, hypoxia‐inducible pathways [20], and extracellular matrix components collectively shape these niches, promoting stemness maintenance and shielding GSCs from therapeutic exposure. These niches also impose distinct metabolic and oxidative constraints, further selecting for cells capable of flexible metabolic adaptation and stress tolerance [21, 22].

From an evolutionary perspective, GSCs represent a cellular strategy optimized for survival rather than maximal proliferation. Their ability to transiently enter quiescent states, repair damage, and later re‐enter proliferative programs confers a selective advantage under repeated therapeutic bottlenecks [23]. This survival‐first strategy distinguishes GSCs from bulk tumor cells and explains why recurrence often arises despite near‐complete radiographic responses.

Taken together, GSCs function as the architectural core of GBM recurrence, integrating transcriptional stemness programs with adaptive stress responses. However, stemness alone cannot fully account for the long‐term persistence of these cells across successive cell divisions. Sustained self‐renewal requires mechanisms that preserve genome integrity and replicative capacity, as well as metabolic programs capable of supporting survival under extreme conditions [24]. These considerations naturally lead to the next pillars of GBM malignancy‐TMMs [4, 25, 26] and metabolic reprogramming [27]—which together cooperate with stemness to establish a durable, therapy‐resistant tumor state [28, 29].

#### 2.1.1 Metabolic Foundations of Stemness in GBM

Stemness in GBM is fundamentally supported by a distinct metabolic architecture that enables GSCs to sustain self‐renewal and resist stress. This section focuses on the core metabolic mechanisms‐including glycolysis, oxidative phosphorylation (OXPHOS), and one‐carbon metabolism [30, 31]—that establish and maintain the stem‐like phenotype of GSCs [32, 33].

GBM is a prototypical example of a metabolically adaptable tumor, and this plasticity is largely driven by the presence of GSCs. These cells, which comprise a small but critical subset within the tumor mass, are endowed with self‐renewal capabilities, multipotency, and intrinsic resistance to conventional therapies. Far from being a static population, GSCs are highly dynamic and capable of metabolic rewiring that supports their stemness under fluctuating environmental conditions. A growing body of evidence indicates that the maintenance of stem‐like properties in GSCs is intimately linked with metabolic reprogramming. In contrast to the classical view that bulk GBM cells predominantly rely on aerobic glycolysis (i.e., the Warburg effect), GSCs exhibit remarkable metabolic flexibility, switching between glycolysis and OXPHOS in a context‐dependent manner [34]. For instance, under normoxic conditions, certain subpopulations of GSCs preferentially utilize OXPHOS, allowing them to efficiently generate ATP and sustain biosynthetic pathways critical for stem cell maintenance [22, 35]. This is supported by studies showing elevated expression of mitochondrial electron transport chain components and mitochondrial biogenesis regulators such as PGC‐1α in OXPHOS‐dominant GSCs [2, 36, 37, 38]. Consistent with this, recent quantitative analyses have demonstrated that PGC‐1α upregulation drives mitochondrial biogenesis and enhances OXPHOS activity in GSCs, reinforcing their metabolic and stemness programs [39].

Conversely, hypoxic microenvironments‐a hallmark of GBM‐favor a glycolytic phenotype in GSCs. Hypoxia‐inducible factors (HIFs—HIF‐1α and HIF‐2α) are stabilized under low oxygen tension, promoting the transcription of genes involved in glycolysis, such as GLUT1 and LDHA, as well as stemness regulators like SOX2 and OCT4 [40]. This co‐activation of glycolytic metabolism and stemness transcriptional programs ensures survival and maintenance of GSCs under stress conditions. Intriguingly, HIF‐2α has been shown to have a more GSC‐specific expression pattern compared to HIF‐1α, linking it more directly to tumor‐propagating capacity. Additionally, GSCs reprogram lipid metabolism to fuel stemness. They display increased uptake and β‐oxidation of fatty acids (FAO), which supports energy homeostasis and redox balance. FAO‐generated NADPH contributes to the detoxification of ROS, which is essential for maintaining genome stability in long‐lived stem‐like cells. Inhibition of key FAO enzymes such as CPT1A has been shown to reduce the tumor‐initiating potential of GSCs, underscoring the functional role of lipid metabolism in stemness maintenance [41]. One‐carbon metabolism, encompassing folate and methionine cycles, is another critical axis co‐opted by GSCs. This pathway supplies essential cofactors for nucleotide synthesis, methylation reactions, and redox control. Elevated activity of serine biosynthesis and folate cycle enzymes has been linked to enhanced self‐renewal and epigenetic regulation in GSCs. For instance, the upregulation of MTHFD2‐a mitochondrial enzyme involved in the folate cycle‐has been associated with poor prognosis and supports DNA replication and repair in proliferating stem‐like cells [42].

Notably, the metabolic state of GSCs is not merely a passive adaptation but plays a causative role in regulating stem cell transcriptional networks. For example, acetyl‐CoA levels derived from glucose and fatty acid metabolism influence histone acetylation, thereby modulating the chromatin landscape to favor stemness gene expression [43, 44]. Similarly, S‐adenosylmethionine (SAM), a product of one‐carbon metabolism, acts as a methyl donor for histone and DNA methylation, further linking metabolism to epigenetic control of the stem‐like state [45]. Another layer of complexity arises from the spatial and temporal heterogeneity of metabolic states within the GBM microenvironment. Recent single‐cell RNA sequencing studies have revealed that GSCs can exist in distinct metabolic states even within the same tumor, with subpopulations exhibiting differential expression of glycolytic, OXPHOS, and FAO‐related gene [46]. These distinct metabolic subtypes correspond to functional states, such as proliferative versus quiescent GSCs, and may dictate sensitivity to therapeutic agents [47, 48].

**FIGURE 1:**
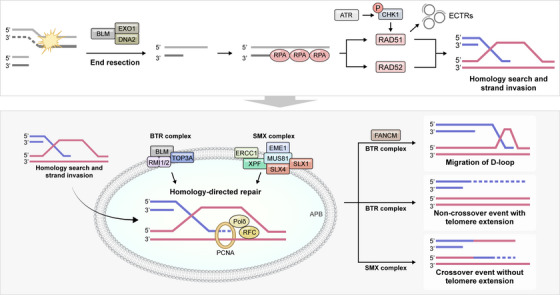
Alternative lengthening of telomeres (ALT) as a telomere maintenance mechanism (TMM) in glioblastoma (GBM). Schematic representation of ALT activation in GBM, in which telomere length is maintained through homologous recombination–based mechanisms in the absence of telomerase, frequently associated with ATRX or DAXX loss. ALT‐positive tumors are characterized by ultra‐long telomeres, ALT‐associated PML bodies (APBs), and extrachromosomal telomeric repeats, and are often linked to genomic instability, mesenchymal or proneural subtypes, and a relatively immunologically active tumor microenvironment, highlighting distinct therapeutic vulnerabilities compared with telomerase‐reactivated GBMs.

In summary, GSCs maintain their stem‐like properties through a tightly regulated and plastic metabolic program that interfaces with transcriptional and epigenetic networks. This metabolic adaptability allows them to thrive under nutrient‐limited, hypoxic, and genotoxic stress conditions, contributing to treatment resistance and tumor recurrence (Figure 1). Therapeutic strategies targeting metabolic vulnerabilities specific to GSCs—such as OXPHOS inhibitors (e.g., metformin, IACS‐010759), FAO blockers, or one‐carbon metabolism disruptors—represent a promising avenue for eradicating the stem‐like reservoir in GBM [49, 50].

### 2.2 TMMs: Enabling Cellular Immortality and Immune Implications and Association With Antigen‐Presenting Cell (APC) Signatures

TMMs are essential for the long‐term proliferation and immortality of GBM cells. Two primary mechanisms‐reactivation of telomerase (typically via TERT promoter mutations) and activation of the alternative lengthening of telomeres (ALT) pathway‐enable tumor cells to circumvent replicative senescence. While TMMs have traditionally been studied in the context of genome stability and cell proliferation, emerging evidence reveals that they also play a significant role in modulating the immune microenvironment of GBM, including the expression of APC signatures and the degree of immune surveillance [51, 52].

The reactivation of telomerase via TERT promoter mutations [53] is the predominant TMM in GBM and is found in more than 80% of primary tumors [54, 55]. Such mutations result in elevated TERT expression, thereby promoting prolonged cellular replicative potential. However, TERT reactivation has also been implicated in immune evasion. Studies have shown that TERT may directly suppress the expression of genes involved in innate immunity and antigen presentation, such as MHC class I, B2M, and TAP1. TERT has also been associated with reduced interferon signaling and dampened NF‐κB activity, contributing to a “cold” immune phenotype in GBM, characterized by low levels of APC infiltration and poor T‐cell activation. In contrast, GBM tumors utilizing the ALT pathway—although less common (seen in ∼5%–15% of cases)—exhibit distinctive genomic and immunological profiles. ALT is typically associated with ATRX/DAXX mutations and involves homologous recombination‐based telomere elongation. Interestingly, ALT‐positive GBMs tend to have increased chromosomal instability, which can lead to the accumulation of cytosolic DNA fragments [56]. These fragments are sensed by innate immune pathways, particularly the cGAS‐STING axis, leading to the activation of type I interferon responses and upregulation of antigen‐presentation machinery [57]. This process may foster a more immune‐inflamed microenvironment, potentially enhancing APC recruitment and T cell priming. Consistent with this hypothesis, transcriptomic analyses from TCGA and single‐cell RNA‐seq datasets have revealed that ALT‐positive GBMs show elevated expression of APC‐related signatures, including HLA‐DRA, CD74, CIITA, and CD86, often co‐expressed in a subset of reactive astrocyte‐like or macrophage‐like cells within the tumor [56].

Mechanistically, ALT‐associated genomic instability promotes the accumulation of cytosolic DNA fragments that activate the cyclic GMP‐AMP synthase (cGAS)STING signaling pathway, resulting in type I interferon responses and induction of antigen‐presentation genes. In addition, ALT‐positive GBMs exhibit elevated reactivation of endogenous retroviral elements (ERVs) and double‐stranded RNA sensing [58], which further stimulate innate immune pathways and upregulate MHC class I/II expression. Together, these findings provide mechanistic evidence that ALT activation not only sustains telomere elongation but also triggers pro‐inflammatory signaling cascades that reshape the tumor immune microenvironment (TME) [59, 60].

Moreover, these tumors tend to have higher infiltration of CD8+ T cells, MHC class II–positive microglia, and dendritic cell (DC)–like populations, suggesting that TMM status could influence immune cell composition [61, 62, 63].

One notable study by Johnson et al. [64] utilized integrative single‐cell analysis across multiple glioma samples and showed that GBMs with low TERT expression and ALT‐like chromatin features were enriched for inflammatory and APC‐related gene expression. This was particularly evident in tumors with hypomethylated TERT promoters or biallelic ATRX loss. These observations support the idea that ALT activity may not only sustain tumor cell immortality but also indirectly modulate immune recognition through the induction of antigen presentation programs. In addition to influencing transcription, telomere abnormalities may impact the immunogenic properties of GBM cells. Telomere attrition and telomeric DNA damage can promote the expression of neoantigens or activate damage‐associated molecular patterns (DAMPs), which in turn can trigger DC maturation and enhance MHC‐mediated antigen presentation. This phenomenon may be more prevalent in ALT+ tumors due to their increased genomic fragility and replication stress [65, 66].

Moreover, differences in epigenetic landscapes at telomeric and subtelomeric regions between ALT‐positive and TERT‐expressing GBMs may affect the activation of ERVs, which serve as strong immunostimulatory signals. Reactivation of ERVs can mimic viral infection, driving interferon responses and MHC upregulation, particularly in ALT‐positive or telomerase‐suppressed GBMs [67]. From a therapeutic standpoint, these findings raise the possibility that ALT‐positive GBMs may be more responsive to immune checkpoint blockade (ICB) or immunotherapies targeting APC–T‐cell interactions. Conversely, TERT+ tumors, with their immunosuppressive epigenetic and transcriptional programs, may require combination approaches to restore immune visibility—such as TERT inhibitors plus STING agonists or epigenetic modulators. Last, telomere length and maintenance status may also shape metabolic features of APCs within the tumor microenvironment. For example, TERT+ GBMs often exhibit hypoxia‐driven metabolic reprogramming that impairs DC activation and antigen presentation through lactate accumulation and acidification of the tumor niche. In contrast, ALT+ tumors, which rely less on glycolysis and more on mitochondrial metabolism, may create a more permissive metabolic landscape for APC function [66, 68, 69].

**FIGURE 2:**
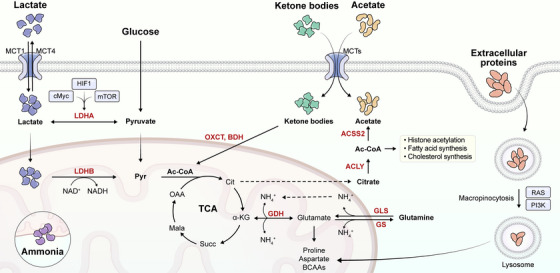
Metabolic reprogramming in GBM stem‐like cells supports tumor growth, survival, and immune evasion. Schematic overview of the major metabolic pathways in glioma stem‐like cells (GSCs), highlighting metabolic plasticity across glycolysis, oxidative phosphorylation (OXPHOS), fatty acid oxidation, one‐carbon metabolism, and glutaminolysis to sustain stemness, biomass synthesis, energy production, and redox homeostasis. Metabolic byproducts such as lactate and kynurenine reshape the tumor immune microenvironment by suppressing T‐cell function and limiting nutrient availability, thereby promoting immune evasion and therapeutic resistance.

In summary, TMMs in GBM (Figure 2) not only dictate replicative immortality but also profoundly influence the tumor‐immune interface. TERT reactivation is associated with immune evasion, suppressed APC activity, and poor immunotherapy response, while ALT activation correlates with higher APC gene expression, increased innate immune signaling, and potentially improved prognosis. These insights underscore the potential of TMM status as both a prognostic biomarker and a stratification tool for immunotherapy in GBM and highlight the need for future studies exploring telomere‐immunity crosstalk in greater mechanistic detail [70, 71].

### 2.3 Metabolic Reprogramming: Fueling the Hostile Tumor Ecosystem and Implications for Tumor‐Immune Crosstalk

Metabolic reprogramming is a hallmark of GBM, not only serving as a driver of tumor growth and survival but also playing a critical role in shaping the TME. GBM cells undergo profound alterations in glucose, lipid, and amino acid metabolism to meet their high biosynthetic and energetic demands. However, this metabolic plasticity also creates a hostile environment for infiltrating immune cells, fundamentally altering their differentiation, effector function, and viability. One of the most notable features of GBM metabolism is its reliance on aerobic glycolysis (the Warburg effect), even under normoxic conditions. As a result, cells exhibit elevated glucose consumption and enhanced lactate generation. Lactate is not merely a metabolic waste product; it acts as a potent immunosuppressive metabolite. High levels of extracellular lactate in the GBM microenvironment impair T‐cell receptor (TCR) signaling, inhibit cytotoxic T lymphocyte (CTL) proliferation, and suppress interferon‐γ (IFN‐γ) secretion. Lactate also polarizes tumor‐associated macrophages (TAMs) toward an M2‐like, anti‐inflammatory phenotype, reducing their capacity to present antigens and produce pro‐inflammatory cytokines such as IL‐12 and TNF‐α [72, 73].

Moreover, GBM cells express high levels of glucose transporters (GLUT1, GLUT3) and consume large amounts of glucose, depriving infiltrating T cells of a critical substrate required for their glycolytic metabolism and effector differentiation [74]. Studies have shown that nutrient competition between tumor cells and immune cells, particularly for glucose and amino acids like tryptophan and arginine, can lead to metabolic exhaustion of CD8+ T cells and natural killer (NK) cells [75]. This competitive deprivation impairs mTOR signaling and diminishes T‐cell cytotoxic function. Tryptophan metabolism is another key axis in GBM‐mediated immune suppression. Indoleamine 2,3‐dioxygenase (IDO), often overexpressed in GBM, catabolizes tryptophan into kynurenine [76, 77]. Kynurenine not only depletes tryptophan levels—critical for T‐cell proliferation—but also acts through the aryl hydrocarbon receptor (AHR) pathway to promote regulatory T‐cell (Treg) differentiation and suppress DC maturation, ultimately dampening antigen presentation and adaptive immune responses [78, 79].

In addition to suppressing T cells, the GBM metabolic landscape reprograms myeloid‐derived suppressor cells (MDSCs) and microglia to adopt immunosuppressive phenotypes. The hypoxic TME, driven by tumor growth outpacing vascular supply, stabilizes HIFs, particularly HIF‐1α, which transcriptionally induces VEGF and arginase‐1. Arginase‐1 depletes arginine, further compromising T‐cell survival and enhancing the suppressive function of MDSCs and M2 macrophages [27]. Of particular interest is the relationship between metabolic programming and APC functionality in GBM. For example, lactate accumulation and hypoxia impair the maturation of DCs, downregulating the expression of MHC class II and co‐stimulatory molecules such as CD80/CD86. This leads to insufficient T cell priming and contributes to the “immune desert” phenotype observed in many GBMs [80]. In addition, lipid accumulation and altered lipid metabolism in tumor‐associated microglia can lead to defective phagocytosis and antigen presentation, favoring immune escape [81]. Targeting metabolic pathways in GBM is thus emerging as a promising strategy to restore immune function. Pharmacologic inhibitors of glycolysis, lactate transport (e.g., MCT1 inhibitors), and IDO, as well as approaches to rewire amino acid metabolism, have shown promise in preclinical models [82]. These interventions aim to not only deprive tumor cells of critical metabolic support but also “recondition” the metabolic microenvironment to be more permissive for immune cell activation and function. Furthermore, there is growing interest in combining metabolic reprogramming agents with immunotherapies, such as immune checkpoint inhibitors (ICIs) or CAR‐T cells [83], to overcome metabolic immune suppression. For instance, strategies to enhance mitochondrial oxidative metabolism in T cells (e.g., via PGC‐1α activation or FAO enhancers) have been proposed to improve T‐cell persistence and cytotoxicity in the hostile GBM TME [27, 84].

**FIGURE 3:**
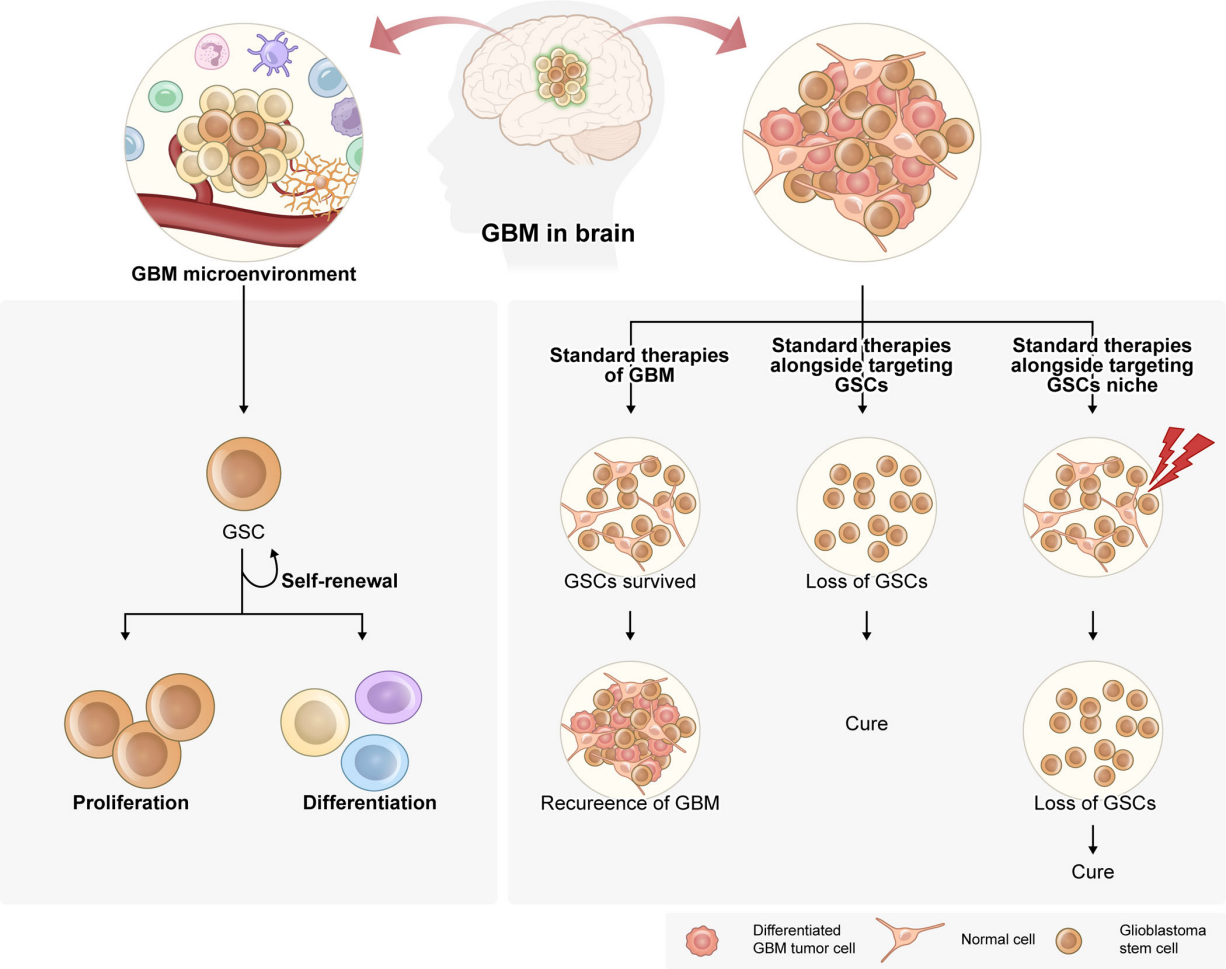
Multifaceted roles of GBM stem‐like cells in tumor maintenance and therapy resistance. Schematic illustration of the central functions of GSCs in GBM, highlighting their self‐renewal capacity, multipotency, and role in sustaining tumor growth, heterogeneity, and therapeutic resistance. GSC stemness is reinforced by core transcriptional regulators, including SOX2, OLIG2, and NANOG, which maintain an undifferentiated, proliferative state and support adaptive responses to metabolic stress and telomere‐associated challenges.

In conclusion, metabolic reprogramming in GBM (Figure 3) is not a cell‐intrinsic phenomenon but a multifaceted regulator of tumor‐immune interactions. By actively shaping the metabolic landscape of the microenvironment, GBM undermines immune surveillance through nutrient competition, immunosuppressive metabolite production, and APC dysfunction. A deeper understanding of these interactions will be critical for designing next‐generation metabolic‐immune combinatorial therapies to improve outcomes in GBM patients [85, 86].

## 3 The Core Nexus: Decoding the Interdependent Triad in GBM

To move beyond compartmentalized models of GBM biology, we introduce the Triadic Nexus as a mechanistically testable framework integrating three core axes: (i) GSC stemness programs, (ii) TMMs (TERT reactivation vs. ALT activation) [53, 87, 88], and (iii) metabolic reprogramming [89]. In this model, these axes are not parallel or independent; instead, they are linked through bidirectional regulatory loops and feed‐forward circuits that collectively sustain tumor persistence. By explicitly defining these interconnections, the Triadic Nexus provides a causal and functional hierarchy through which diverse molecular observations can be unified into a coherent biological model [30, 90, 91, 92].

In the following subsections, we systematically dissect each dimension of the Triadic Nexus. We first examine how stemness is both shaped by and dependent on metabolic plasticity (Nexus I), followed by the reciprocal relationship between stemness and telomere stability (Nexus II) [93, 94, 95, 96, 97]. We then discuss how metabolic states directly regulate telomere maintenance and telomere‐associated stress responses (Nexus III). These pairwise interactions are finally synthesized into an integrated model describing a self‐reinforcing, therapy‐resistant loop that underlies GBM persistence.

### 3.1 Nexus I: Stemness Fuels and Is Fueled by Metabolic Plasticity

GBM exhibits remarkable intratumoral heterogeneity, largely attributable to a subset of GSCs. These cells exhibit high tumorigenic potential, self‐renewal capacity, resistance to therapy, and the ability to regenerate tumor bulk after treatment. Mounting evidence suggests that the properties of stemness in GSCs are intimately linked to both TMMs and metabolic reprogramming, forming a tightly integrated triad that sustains tumor progression and therapy evasion [10, 98, 99].

Historically, GSC biology has been conceptualized through discrete frameworks: stemness as a transcriptional hierarchy (e.g., SOX2, OLIG2, NANOG), telomere maintenance as a proliferative safeguard via TERT or ALT, and metabolism as a supportive process enabling energy and redox balance. These domains were largely studied in isolation, with limited understanding of their interdependence [25, 100, 101].

In contrast, our Triadic Nexus model posits that stemness, telomere regulation, and metabolic reprogramming constitute an interlocked, self‐reinforcing system [102]. In this framework, telomere dynamics serve not only as a proliferation checkpoint but also as a metabolic sensor influencing redox and epigenetic states, while metabolic cues reciprocally regulate telomerase activity and stemness maintenance. This integrated model provides a mechanistically testable hypothesis—that disrupting one axis (e.g., telomerase or FAO inhibition) induces compensatory stress across the others, revealing synthetic vulnerabilities exploitable for combinatorial therapy in GBM [103, 104] (Table 1).

**TABLE 1:** Comparison between traditional glioma stem‐like cell (GSC) models and the proposed Triadic Nexus framework.

Aspect	Traditional GSC model	Triadic Nexus model (this review)
Conceptual view	Independent modules: Stemness, telomere biology, and metabolism studied separately [105, 106]	Integrated, dynamic triad with bidirectional regulation among all three components
Role of telomere maintenance	Ensures replicative immortality only [4, 28, 55, 56, 107]	Acts as both genomic safeguard and metabolic/epigenetic regulator
Metabolic reprogramming	Supports energy production and ROS control [108, 109]	Serves as a driver and sensor of stemness and telomere activity
Stemness regulation	Transcription‐factor–driven hierarchy [15, 110]	Coupled to telomere status and metabolic flux through redox/epigenetic feedback
Testable prediction	Each axis targeted individually	Perturbation of one arm (e.g., telomerase inhibition) triggers measurable shifts in the others
Therapeutic implication	Monotherapy targeting single pathway	Rational combinatorial therapy exploiting triad interdependence

Unlike traditional models that treat stemness, telomere biology, and metabolism as discrete hallmarks, the Triadic Nexus emphasizes their dynamic interdependence. This integrative perspective not only reconciles disparate findings across molecular, metabolic, and immune studies but also generates testable predictions: perturbation of any single axis is expected to propagate compensatory stress across the others. Such systemlevel behavior distinguishes the Triadic Nexus from descriptive frameworks and positions it as a hypothesis‐driven model with direct therapeutic implications.

While the previous section outlines the core metabolic mechanisms underlying stemness, this section explores the dynamic adaptability of GSCs in response to environmental cues. Here, we discuss how GSCs flexibly shift between glycolysis, OXPHOS, and fatty acid oxidation to survive in hypoxic, nutrient‐limited, or therapyinduced stress conditions, and how this metabolic flexibility contributes to therapeutic resistance.

**FIGURE 4:**
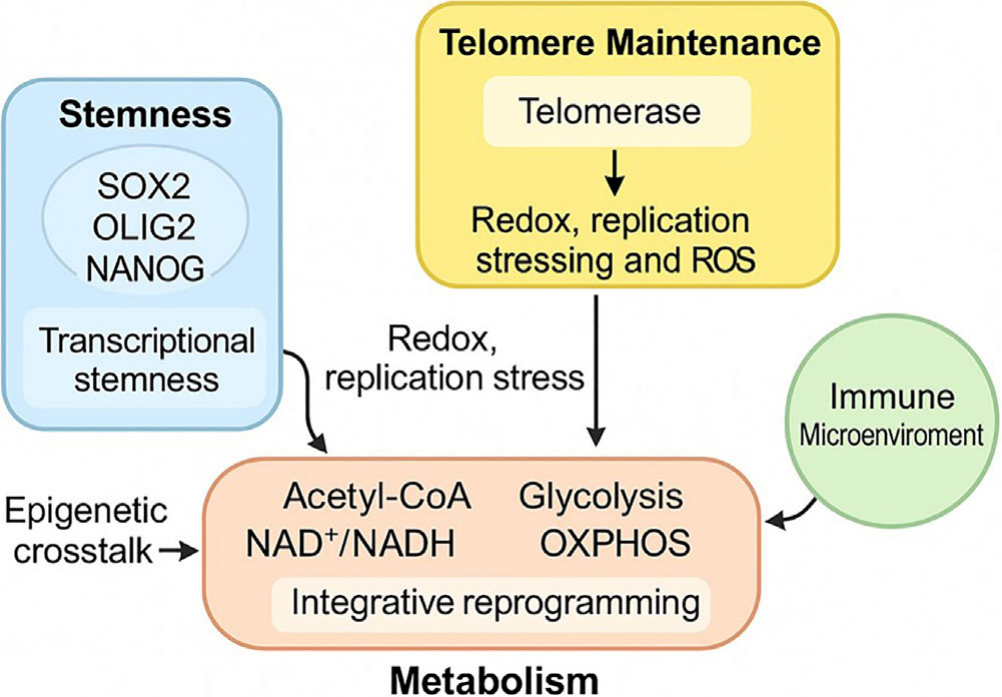
Integrated regulatory network linking stemness, telomere maintenance, metabolism, and immune signaling in GBM stem‐like cells. Schematic depiction of the Triadic Nexus model, illustrating bidirectional feedback among transcriptional stemness programs (SOX2, OLIG2, NANOG), TMMs (telomerase or ALT‐associated replication and redox stress), and metabolic reprogramming (glycolysis, OXPHOS, and key metabolic cofactors). Telomere‐ and metabolism‐derived signals converge on epigenetic and immune regulatory pathways, shaping immune suppression or activation and sustaining GSC plasticity and therapeutic resistance.

Metabolic rewiring is central to GSC function [22]. Unlike differentiated tumor cells that rely heavily on glycolysis, GSCs display remarkable metabolic flexibility. Depending on the microenvironmental context—such as hypoxia, nutrient availability, and redox balance—GSCs can toggle between glycolysis, OXPHOS, and fatty acid oxidation (FAO). This adaptability supports their survival in diverse niches, including perivascular regions and hypoxic tumor cores. For example, GSCs (Figure 4) enriched in perinecrotic zones preferentially utilize OXPHOS, maintaining low ROS levels and resisting hypoxia‐induced cell death. Conversely, GSCs in nutrient‐rich, normoxic areas may exploit glycolysis and de novo lipogenesis for rapid proliferation. Transcriptional regulators such as c‐MYC, PGC‐1α, and HIF‐1α orchestrate this metabolic shift, linking metabolic programs to stemness gene expression (e.g., SOX2, NANOG, OLIG2).

Furthermore, metabolism intersects with epigenetic regulation of stemness. α‐Ketoglutarate (α‐KG) and other TCA cycle intermediates influence the activity of histone demethylases (e.g., JmjC domain‐containing proteins) and DNA demethylases (e.g., TETs), thereby modulating chromatin accessibility at stemness‐associated loci. This metabolic‐epigenetic feedback loop contributes to the maintenance of the GSC phenotype and its capacity for differentiation [111, 112].

### 3.2 Nexus II: Stemness Is Co‐Dependent With Telomere Stability

Telomere integrity is a prerequisite for the sustained proliferative capacity of GSCs. In contrast to most normal somatic cells, which undergo replicative senescence due to telomere shortening, GSCs evade this fate by activating TMMs. Two major TMMs have been identified in GBM: telomerase activation and ALT. The telomerase reverse transcriptase (TERT) promoter mutation (e.g., C228T and C250T) is prevalent in the classical and proneural GBM subtypes and leads to enhanced TERT expression. This activation not only maintains telomere length but also confers non‐canonical pro‐stemness functions, such as regulation of WNT/β‐catenin and NF‐κB signaling pathways [113, 114].

Conversely, a subset of GBMs, particularly those with ATRX or DAXX mutations, utilize the ALT mechanism, characterized by homologous recombination‐based elongation of telomeres. Notably, ALT‐positive GBMs are often found in IDH‐mutant, TP53‐mutant tumors and tend to exhibit less aggressive progression and better prognosis. Interestingly, recent studies have shown that ALT activity may constrain the stem‐like phenotype by limiting excessive proliferation and reducing oxidative stress, thereby favoring a more quiescent stem cell state.

While ALT activity has been proposed to constrain excessive proliferation by maintaining redox balance and promoting genomic stability in quiescent GSCs, emerging evidence also suggests that ALT can support proliferative signaling under oncogenic or DNA damage–induced stress by sustaining telomere length through recombination‐based elongation. Thus, the functional impact of ALT on stemness and proliferation appears to be context‐dependent, dynamically shaped by tumor microenvironmental and metabolic conditions.

These observations suggest that TMMs not only support replicative immortality but also influence the differentiation and plasticity of GSCs [51, 115, 116, 117].

### 3.3 Nexus III: Metabolism Directly Powers and Regulates Telomere Maintenance

Emerging studies highlight direct links between metabolism and telomere regulation in GSCs. For instance, telomerase activity is modulated by cellular redox states and nutrient‐sensing pathways such as mTOR and AMPK [118, 119]. High glycolytic flux provides ATP and NADPH needed for telomere elongation and TERT stabilization [120, 121]. In addition, glutamine metabolism, a key anaplerotic input for the TCA cycle, is essential for ALT pathway activation by supplying nucleotide precursors and maintaining redox homeostasis during DNA recombination events at telomeres [95, 122, 123, 124].

Moreover, the metabolic stress associated with hypoxia and ROS accumulation may favor ALT activation by promoting DNA damage and homologous recombination machinery. Interestingly, ALT‐positive GSCs exhibit increased mitochondrial biogenesis and oxidative stress response genes, suggesting a compensatory reliance on mitochondrial metabolism. This observation opens potential avenues for selectively targeting ALT‐driven tumors with mitochondrial disruptors [125, 126].

### 3.4 Synthesizing the Model: A Self‐Reinforcing, Therapy‐Resistant Loop

Collectively, the interactions described in Nexus I–III converge into a unified, self‐reinforcing regulatory loop that underlies GBM persistence and therapeutic failure. In this integrated model, GSC stemness [99], TMMs, and metabolic reprogramming do not function as parallel hallmarks but instead form a dynamically coupled system in which perturbation of one axis propagates adaptive responses across the others [127]. Metabolic plasticity sustains stemness by shaping epigenetic and redox landscapes, while stemness programs actively rewire metabolic pathway selection to favor survival under stress. In parallel, telomere maintenance acts as both a permissive factor for long‐term self‐renewal and a molecular sensor that links replication stress, redox balance, and mitochondrial signaling [128, 129, 130, 131].

This triadic coupling generates a feed‐forward architecture that buffers GSCs against therapeutic pressure. Cytotoxic or metabolic stress induces compensatory shifts‐such as enhanced mitochondrial metabolism, altered telomerase or ALT activity, and reactivation of stemness‐associated transcriptional programs—that collectively preserve tumor‐propagating capacity. As a result, therapies targeting a single pathway often fail not because the target is dispensable but because the remaining axes dynamically adapt to restore system‐level equilibrium [132, 133, 134, 135].

Importantly, this self‐reinforcing loop provides a mechanistically testable framework for understanding therapy resistance. The Triadic Nexus predicts that effective disruption of GBM persistence will require coordinated intervention across multiple axes, exploiting moments of synthetic vulnerability created when compensatory capacity is exceeded. By explicitly defining how stemness, telomere biology, and metabolism are functionally integrated, this model moves beyond descriptive associations and establishes a systems‐level logic for rational combinatorial therapy design [136, 137].

## 4 Translational Implications: Exploiting the Nexus for Therapeutic Intervention

### 4.1 Targeting GSC Stemness Pathways: Current Inhibitors and Challenges

Given the central role of GSCs in tumor propagation and recurrence, substantial efforts have been devoted to directly targeting stemness‐associated signaling pathways in GBM. Canonical developmental pathways, including NOTCH, WNT/β‐catenin, Hedgehog (SHH), and STAT3 signaling, have been identified as key regulators of GSC self‐renewal, maintenance, and resistance to differentiation. Accordingly, multiple pharmacologic inhibitors‐such as γ‐secretase inhibitors targeting NOTCH signaling, WNT pathway antagonists, SHH pathway inhibitors, and small‐molecule STAT3 inhibitors‐have been evaluated in preclinical models and early‐phase clinical studies [18, 138, 139, 140].

Despite a strong mechanistic rationale, the clinical efficacy of direct stemness pathway inhibition has been limited. One major challenge is pathway redundancy and compensatory signaling: suppression of a single stemness pathway often leads to rapid activation of parallel networks that preserve GSC identity. In addition, GSCs exhibit profound phenotypic plasticity, allowing differentiated tumor cells to reacquire stem‐like properties following pathway inhibition, thereby undermining durable therapeutic responses. These adaptive state transitions are further reinforced by protective tumor niches, such as hypoxic and perivascular regions, which buffer GSCs from pharmacologic stress [141].

Another critical limitation is toxicity. Many stemness pathways are evolutionarily conserved and essential for normal tissue homeostasis, particularly in the nervous system. As a result, systemic inhibition frequently produces dose‐limiting adverse effects, restricting the therapeutic window and precluding sustained target suppression in patients. Furthermore, transcription‐factor–driven stemness programs are inherently difficult to inhibit directly, as they often lack well‐defined enzymatic pockets amenable to small‐molecule targeting.

Collectively, these challenges highlight a fundamental limitation of strategies that aim to eradicate GSCs by targeting stemness pathways in isolation. Rather than functioning as autonomous signaling modules, stemness programs are embedded within a broader adaptive system that includes metabolic reprogramming and telomere maintenance. This realization has prompted a conceptual shift away from single‐pathway stemness inhibition toward therapeutic approaches that exploit the interdependence between stemness, metabolism, and genome maintenance mechanisms—a framework that is elaborated in the following sections [142, 143].

### 4.2 Attacking Telomere Maintenance: Telomerase Inhibitors, ALT‐Targeting Strategies, and Inducing Telomere Crisis

Telomere maintenance constitutes a fundamental dependency in GBM, particularly in GSCs, which require sustained replicative capacity to enable long‐term tumor propagation and recurrence. Disruption of TMMs therefore represents a rational strategy to undermine cellular immortality and destabilize the stemness programs that fuel therapeutic resistance.

In telomerase‐positive GBMs, pharmacologic inhibition of telomerase has been shown to impair tumor growth not only through progressive telomere shortening but also via broader effects on stemness‐associated signaling networks. Telomerase activity intersects with pathways governing self‐renewal and transcriptional plasticity, positioning telomerase as an active participant in the maintenance of GSC identity rather than a passive regulator of chromosome ends. Accordingly, telomerase inhibition can simultaneously constrain proliferative potential and weaken stemness‐supportive circuitry.

The convergence of stemness, metabolism, and telomere maintenance represents a central vulnerability in GBM. Targeting one node of this triad can potentially perturb the others. For instance, inhibitors of telomerase (e.g., imetelstat) not only shorten telomeres but also attenuate WNT‐mediated stemness pathways. Similarly, OXPHOS inhibitors (e.g., IACS‐010759) and FAO blockers impair GSC survival and sensitize them to radiotherapy. Combination strategies that exploit these interdependencies‐for example, dual inhibition of telomerase and mitochondrial metabolism‐are under active investigation. Furthermore, understanding the metabolic signatures associated with different TMMs could enable precision therapy. For example, ALT‐positive tumors may be less glycolytic and more reliant on mitochondrial function, making them susceptible to oxidative stress inducers or mitochondrial translation inhibitors. At the same time, stemness signatures could serve as biomarkers for stratifying patients likely to benefit from metabolism‐ or telomere‐targeting approaches [144, 145].

As a concrete example, combining the PARP inhibitor olaparib with standard radiotherapy ± temozolomide (guided by MGMT status) has a mechanistic and pharmacokinetic rationale in GBM and is being explored clinically; concurrent olaparib during RT followed by maintenance olaparib with adjuvant TMZ has shown feasibility in early‐phase studies (PARADIGM‐2, OPARATIC, OLA‐TMZ‐RTE‐01) [146, 147].

Complementing telomerase‐directed strategies, increasing attention has been directed toward tumors that maintain telomeres through ALT, a recombination‐based mechanism frequently associated with ATRX or DAXX loss. ALT‐positive GBMs exhibit heightened dependence on homologous recombination, replication stress tolerance, and DNA damage response pathways [148, 149]. Therapeutic approaches that exacerbate telomeric DNA damage or disrupt ALT‐associated repair processes therefore hold promise for selectively destabilizing telomere integrity in this molecular subtype [101, 150].

Beyond direct inhibition of telomere elongation, an emerging therapeutic concept involves deliberately inducing telomere crisis by overwhelming the capacity of tumor cells to preserve telomere integrity. In this context, targeting genome maintenance pathways that intersect with telomere biology has shown particular promise. As a concrete clinical example, combining the PARP inhibitor olaparib with standard radiotherapy, with or without temozolomide guided by MGMT promoter status, is supported by strong mechanistic and pharmacokinetic rationale in GBM. Early‐phase clinical studies evaluating concurrent PARP inhibition during radiotherapy followed by maintenance olaparib with adjuvant temozolomide have demonstrated feasibility, illustrating how disruption of DNA repair and telomere‐associated genome stability can synergize with cytotoxic stress to limit tumor regrowth [133, 151, 152, 153].

Collectively, these observations position telomere maintenance as a central hub within a self‐reinforcing adaptive network that integrates genome stability, metabolic plasticity, and stemness. Therapeutic strategies that attack telomerase activity, exploit ALT‐specific vulnerabilities, or induce telomere crisis‐particularly when combined with metabolic or cytotoxic stress‐offer a rational means to push GBM cells beyond their adaptive limits and achieve more durable treatment responses [154, 155, 156].

As summarized in Table 2, TMMs define distinct metabolic dependencies and therapeutic vulnerabilities in glioma.

**TABLE 2:** Telomere maintenance mechanism (TMM)—Stratified metabolic vulnerabilities and rational combination strategies in glioma.

TMM subtype	Dominant metabolic/vulnerability axis	Proposed combination therapy	Sequence/dosing principle	Representative trial/reference
**TERT^+^ (telomerase reactivation)**	Glycolysis‐dominant metabolism with lactate‐driven immunosuppression; telomerase dependency	hTERT‐targeting immunotherapy (DNA vaccine) + PD‐1 blockade + standard RT/TMZ | (alternative axis) IDO pathway inhibition + RT/TMZ	Concurrent priming during RT/TMZ to enhance tumor antigen presentation, followed by immune checkpoint blockade to sustain cytotoxic T‐cell activity	**NCT03491683** (INO‐5401/INO‐9012 + cemiplimab + RT/TMZ; INO‐5401 includes hTERT) **NCT02052648** (indoximod + TMZ in malignant glioma)
**ALT^+^ (ATRX/DAXX loss)**	Replication stress and DNA damage response (DDR) dependency; ALT–ATR axis vulnerability (with secondary redox/OXPHOS stress)	ATR inhibition combined with DNA‐damaging therapy (radiotherapy or alkylating agents); immunotherapy as a downstream consolidation strategy	Sequential or concurrent induction of DNA damage followed by ATR blockade to prevent repair and promote synthetic lethality	Mechanistic rationale for ATR dependency in ALT tumors; translational relevance reviewed in **Waitkus et al. [70]**; ATR inhibitor berzosertib evaluated in ATRX‐altered solid tumors (**NCT03718091**)
*(Exploratory)* Telomerase inhibition	Telomerase catalytic activity	Telomerase inhibitor (imetelstat) ± standard therapy (investigational)		Pediatric recurrent CNS tumors including high‐grade glioma (**NCT01836549**)

Data sources: ClinicalTrials.gov.

TERT‐reactivated tumors preferentially exploit glycolytic metabolism and lactate‐driven immunosuppressive circuits, rendering them amenable to combination strategies that integrate telomerase‐directed immunotherapy with ICB and standard chemoradiation. Consistent with this concept, clinical trials incorporating hTERT‐containing DNA vaccines together with PD‐1 inhibition during radiotherapy and temozolomide have demonstrated the feasibility of therapeutically targeting telomerase dependency in GBM (e.g., NCT03491683).

In contrast, ALT‐positive gliomas, frequently associated with ATRX or DAXX loss, exhibit heightened replication stress and a pronounced reliance on the ATR‐mediated DNA damage response. This dependency creates a rational therapeutic window for ATR inhibition in combination with DNA‐damaging modalities such as radiotherapy or alkylating agents. Although ALT‐selective metabolic targeting remains largely preclinical, the underlying biological framework and therapeutic implications of ALT‐associated vulnerabilities have been comprehensively reviewed by Waitkus et al. [157]. Collectively, these observations support a TMM–stratified framework for the rational design of combination therapies in glioma.

### 4.3 Disrupting Metabolic Dependencies: Targeting Glycolysis, Glutaminolysis, and Redox Homeostasis

Metabolic reprogramming is a defining feature of GBM and constitutes a core adaptive mechanism that supports tumor growth, therapy resistance, and immune evasion. In GSCs, metabolic plasticity extends beyond bioenergetic adaptation to actively sustain stemness programs, preserve telomere integrity, and buffer oxidative and genotoxic stress. These dependencies render metabolic pathways attractive therapeutic targets for destabilizing the adaptive resilience of GBM.

Glycolysis represents a primary metabolic axis exploited by many GBMs, providing rapid ATP generation and metabolic intermediates required for nucleotide synthesis, lipid production, and epigenetic regulation [158]. Elevated glycolytic flux also contributes to acidification of the tumor microenvironment through lactate production, promoting immune suppression and invasive behavior. In GSCs, glycolysis has been linked to maintenance of stemness‐associated transcriptional states and resistance to oxidative damage. Pharmacologic inhibition of glycolysis or glucose transport can therefore impair self‐renewal capacity and enhance sensitivity to radiotherapy, although compensatory metabolic rewiring often limits the durability of single‐agent responses [159].

Glutaminolysis constitutes a second major metabolic dependency, supplying carbon and nitrogen to fuel the tricarboxylic acid (TCA) cycle, nucleotide biosynthesis, and redox balance [160]. GSCs frequently exhibit heightened reliance on glutamine metabolism to sustain mitochondrial function and antioxidant defenses. Disruption of glutamine uptake or glutaminase activity can compromise anaplerotic flux, increase ROS accumulation, and selectively impair tumor cell viability, particularly under hypoxic or treatment‐induced stress conditions [161, 162].

Maintenance of redox homeostasis represents a critical downstream consequence of altered glycolysis and glutaminolysis. GBM cells operate near the threshold of oxidative tolerance, balancing elevated ROS production with robust antioxidant systems supported by NADPH‐generating pathways [163]. Perturbation of redox balance—either through inhibition of antioxidant capacity or forced ROS accumulation—can preferentially target GSCs, which rely on tightly regulated redox control to preserve genomic stability and stemness under stress [164, 165].

Beyond single‐pathway interventions, a promising therapeutic direction lies in disrupting key crosstalk hubs that integrate stemness, telomere maintenance, and metabolic signaling. For example, the TERT–NF‐κB–PGC1α axis links telomere reactivation with mitochondrial biogenesis and redox regulation, representing a strategic target for combined inhibition. Similarly, co‐targeting HIF‐2α–IDH1 metabolic coupling could simultaneously reduce stemness maintenance and suppress hypoxia‐driven immunosuppression [20]. Moreover, integrating immunometabolic reprogramming‐such as lactate transport blockade (MCT1 inhibitors) or kynurenine‐AHR pathway inhibition‐with telomere‐ or metabolism‐targeted therapies could recondition the GBM microenvironment and enhance responsiveness to ICB [166, 167, 168]. Future clinical translation will likely depend on adaptive trial designs that align molecular subtype classification (TERT^+^ vs. ALT^+^) with dynamic biomarkers of metabolic stress [117, 169], allowing precision‐guided, multi‐target combination strategies to overcome the intrinsic resilience of GBM [170, 171, 172, 173].

Collectively, these findings underscore metabolism not merely as a downstream consequence of oncogenic signaling but as a central integrator of stemness, telomere maintenance, and immune modulation. Therapeutic strategies that disrupt glycolysis, glutaminolysis, and redox homeostasis—particularly when deployed in combination and guided by molecular and metabolic biomarkers—offer a rational means to collapse the self‐reinforcing adaptive networks that sustain GBM persistence. By targeting metabolic dependencies within the broader Triadic Nexus, it may be possible to enhance therapeutic durability and overcome the intrinsic resistance that has long limited clinical progress in GBM.

### 4.4 Rational Combinatorial Therapies: The Future Lies in Simultaneously Targeting Multiple Nodes of the Nexus to Prevent Escape and Resistance

The persistent failure of single‐agent therapies in GBM underscores a fundamental principle: Adaptive escape is not an exception but an intrinsic property of the disease. Within the Triadic Nexus, stemness, metabolic plasticity, and telomere maintenance form a coupled adaptive system capable of rebalancing itself when any single node is perturbed. Rational combinatorial therapy therefore demands simultaneous disruption of multiple nexus nodes to exceed the compensatory capacity of tumor cells and prevent state switching that fuels recurrence [174, 175].

A key design principle of nexus‐based combination therapy is orthogonality—the concurrent targeting of functionally distinct yet interdependent processes. Rather than layering agents with overlapping mechanisms, effective combinations should impose non‐redundant constraints on replication, energy metabolism, redox balance, and genome maintenance. By collapsing multiple adaptive degrees of freedom at once, such strategies aim to convert transient stress responses into irreversible loss of tumor‐propagating capacity [176, 177, 178].

Equally important is temporal coordination. The sequence and timing of therapeutic interventions can critically shape adaptive trajectories. For example, priming tumors with metabolic or telomere‐directed stress may restrict the ability of GSCs to activate repair and survival programs in response to subsequent cytotoxic or immune‐based therapies [179]. Conversely, poorly timed combinations risk enabling compensatory rewiring before system‐level collapse is achieved. Rational scheduling should therefore be guided by dynamic biomarkers that reflect real‐time metabolic stress, telomere dysfunction, and stemness state [180].

A third principle involves context‐aware patient stratification. The Triadic Nexus is not uniformly configured across all GBMs; instead, tumors occupy distinct adaptive states shaped by TMMs, metabolic preferences, and stemness programs. Combinatorial strategies must therefore be aligned with molecular context, selecting targets that exploit dominant dependencies rather than applying uniform regimens. This approach shifts therapeutic logic from population‐averaged treatment to state‐specific intervention.

Finally, effective combinatorial therapy must account for the tumor microenvironment as an active participant in resistance. Metabolic competition, hypoxia, and immunosuppressive signaling provide external buffering that can blunt otherwise potent intracellular targeting. Combinations that simultaneously disrupt tumor‐intrinsic nexus nodes and microenvironmental support systems are more likely to achieve durable control than strategies confined to cancer cells alone [75, 181, 182].

Taken together, rational combinatorial therapies informed by the Triadic Nexus move beyond empirical drug pairing toward principled system‐level intervention. By simultaneously constraining multiple adaptive axes, coordinating temporal deployment, and aligning treatment with tumor state, this framework offers a path to overcoming escape and resistance that have long defined GBM treatment failure.

### 4.5 Biomarkers and Patient Stratification: Using TMM and Metabolic Signatures to Guide Therapy

Effective translation of nexus‐based therapeutic strategies in GBM requires robust biomarkers that can stratify patients according to dominant biological dependencies. Given the intrinsic heterogeneity and plasticity of GBM, static genetic alterations alone are insufficient to guide treatment selection. Instead, functional biomarkers reflecting TMMs and metabolic state offer a more informative framework for patient stratification and precision therapy.

TMM status represents a foundational axis for classification. Tumors driven by telomerase reactivation (TERT^+^) and those relying on ALT (ALT^+^) exhibit distinct biological behaviors, stress responses, and therapeutic susceptibilities. As such, TMM classification provides an initial stratification layer that informs downstream vulnerabilities without presupposing specific therapeutic agents. Importantly, TMM status is relatively stable, compared with transcriptional states, making it a practical anchor for patient grouping.

Building upon TMM classification, metabolic signatures offer a second, orthogonal dimension for stratification. GBMs occupy a spectrum of metabolic states ranging from glycolysis‐dominant to mitochondrial‐ and oxidative metabolism‐dependent phenotypes. These states influence redox balance, stress tolerance, and interaction with the tumor microenvironment. Metabolic profiling—derived from transcriptomic, metabolomic, or imaging‐based readouts—can therefore identify dominant energy and redox dependencies that are not captured by genomic markers alone.

Stemness‐associated transcriptional programs provide an additional layer of functional stratification. Rather than serving as binary markers, stemness signatures reflect the probability that a tumor harbors therapy‐resistant, tumor‐propagating cell states. Integrating stemness scores with TMM and metabolic features allows identification of tumors that are most likely to exhibit adaptive escape following single‐modality treatment and may therefore require intensified or multi‐node therapeutic strategies.

Critically, biomarker‐guided stratification in GBM must account for temporal dynamics. Metabolic state, redox stress, and stemness are not fixed properties but fluctuate in response to therapy and microenvironmental pressure. Longitudinal assessment using dynamic biomarkers—such as stress‐response signatures, metabolic flux indicators, or circulating tumor‐derived markers—may enable real‐time adjustment of therapeutic strategy, aligning with adaptive trial designs rather than rigid treatment algorithms [183, 184, 185].

Together, these considerations support a multi‐layered stratification framework in which TMM status defines foundational tumor identity, metabolic signatures reveal dominant adaptive dependencies, and stemness programs indicate the likelihood of therapeutic escape [186]. By integrating these axes, patient stratification can move beyond descriptive classification toward actionable state‐based decision‐making [187]. Such an approach is essential for deploying nexus‐informed therapies with maximal precision and for avoiding overtreatment or ineffective combinations in a disease defined by adaptability.

## 5 TMM‐Defined GSC Subtypes: Molecular Signatures, Metabolic Dependencies, and Therapeutic Vulnerabilities

Rationale. Distinct TMMs in GBM‐TERT reactivation versus ALT activation‐shape non‐overlapping GSC subtypes with characteristic immune and metabolic phenotypes that are therapeutically actionable.

### 5.1 TERT^+^ GSC Subtype

The TERT^+^ GSC subtype is characterized by recurrent TERT promoter mutations (most frequently C228T and C250T), with preservation of ATRX and DAXX function. These genomic alterations drive strong telomerase reactivation and establish an epigenetic program linked to immune suppression and the control of endogenous retroviral (ERV) elements. At the transcriptomic and immune level, TERT^+^ GSCs display downregulation of antigen‐presentation machinery‐including MHC‐I, TAP, and B2M‐along with attenuated interferon signaling, which collectively create a “cold” tumor microenvironment (TME) characterized by reduced APC activity [188, 189, 190, 191]. Metabolically, these cells exhibit high glycolytic flux and lactate accumulation, facilitated by monocarboxylate transporters (MCTs), and depend heavily on the tryptophan‐IDO‐kynurenine‐AHR axis, which promotes Treg polarization and dendritic‐cell suppression, further reinforcing immune evasion [192]. Consequently, TERT^+^ GSCs show specific therapeutic vulnerabilities: They are potentially responsive to telomerase inhibitors and may benefit from epigenetic or STING agonists that restore immune visibility. In parallel, glycolysis or lactate‐transport blockade and IDO/AHR pathway inhibition, especially in combination with immune‐checkpoint blockade, represent promising strategies to overcome their metabolic and immunologic resistance [58, 170, 173, 193].

### 5.2 ALT^+^ GSC Subtype

The ALT^+^ GSC subtype is defined by loss of ATRX and DAXX, which leads to the activation of the ALT pathway. These tumors exhibit ultra‐long telomeres, the formation of ALT‐associated PML bodies (APBs), and elevated levels of extrachromosomal telomeric repeats (ECTRs). Such genomic instability contributes to chromosomal fragility and triggers cGAS–STING pathway activation, linking telomere dysfunction to innate immune sensing. At the transcriptomic and immune level, ALT^+^ GSCs display upregulation of APC gene signatures—including HLA‐DRA, CD74, CIITA, and CD86—and enhanced innate immune activation accompanied by increased CD8^+^ T‐cell infiltration, resulting in a comparatively “hotter” immune microenvironment than that of TERT^+^ tumors.

From a metabolic perspective, ALT^+^ GSCs rely more heavily on mitochondrial metabolism and OXPHOS, sustained by glutamine anaplerosis and redox stress–response pathways that support recombination‐based telomere elongation and maintain genomic stability under oxidative stress. These features give rise to unique therapeutic vulnerabilities: mitochondrial translation and OXPHOS inhibitors, as well as oxidative‐stress–inducing agents or innate immune agonists (such as STING activators), can effectively exploit the ALT‐associated DNA damage response. Moreover, the immune‐inflamed phenotype of ALT^+^ tumors suggests a greater potential responsiveness to ICIs. Collectively, these observations support the concept of a TMM‐anchored taxonomy of GSCs—a mechanistically testable and clinically meaningful classification that can guide rational combinatorial therapies co‐targeting telomere biology, tumor metabolism, and immune regulation. These findings underscore that stemness, telomere maintenance, and metabolic reprogramming are not isolated phenomena but are dynamically interwoven through bidirectional regulatory circuits that sustain GBM stem‐like cell (GSC) identity, therapeutic resistance, and immune adaptation. Metabolic intermediates such as acetyl‐CoA, NAD^+^/NADH, and SAM influence epigenetic remodeling of stemness genes (e.g., SOX2, OLIG2, NANOG) [194, 195], while stemness‐associated transcriptional programs reciprocally modulate both telomerase activity and metabolic pathway selection (e.g., glycolysis vs. OXPHOS) [5, 196]. In parallel, telomere dynamics act as a molecular sensor linking replication stress, redox balance, and mitochondrial signaling, thereby integrating genome stability with metabolic state [43, 197]. This Triadic Nexus forms a self‐reinforcing feedback network in which perturbation of any single axis propagates compensatory changes across the others—a property that may be exploited for rational combinatorial therapies targeting telomere biology, metabolism, and stemness simultaneously [79, 198, 199] (Figure 4).

Recent single‐cell multi‐omics analyses [200] have begun to uncover direct evidence linking TMMs with metabolic programs at the cellular level in GBM. Within individual tumors, subpopulations of GSCs exhibit distinct TMM–metabolic states‐for instance, TERT‐high, glycolytic clusters versus ALT‐like, OXPHOS‐enriched clusters—highlighting metabolic heterogeneity tightly coupled to telomere regulation [201, 202]. These patterns suggest that TMM status shapes [26] not only replicative potential but also metabolic dependencies and immune interactions. Under therapeutic stress, such as radiotherapy or temozolomide treatment, single‐cell trajectories indicate dynamic switching between TMM and metabolic states, potentially enabling adaptive resistance [14]. Thus, integrating single‐cell evidence of TMM–metabolism coupling and intratumoral heterogeneity provides a framework for understanding how the telomere‐metabolism nexus may evolve under therapy and drive tumor persistence [32, 203, 204].

## 6 Conclusion and Future Perspectives

### 6.1 Summary of the Integrated Model

In this review, we propose the Triadic Nexus as an integrated framework to explain the persistence, plasticity, and therapeutic resistance of GBM. Rather than functioning as independent hallmarks, GSC stemness, TMMs, and metabolic reprogramming form a dynamically coupled system that sustains tumor propagation under therapeutic pressure. Within this nexus, stemness programs dictate adaptive state transitions, telomere maintenance preserves long‐term replicative potential, and metabolic plasticity provides the energetic and redox flexibility required for survival in hostile microenvironments.

Crucially, this model reconciles diverse molecular, metabolic, and immunologic observations into a coherent biological logic. Telomere dynamics emerge not merely as a safeguard against replicative senescence but as a molecular sensor that integrates replication stress, redox balance, and mitochondrial signaling. Metabolic intermediates shape epigenetic regulation of stemness‐associated genes, while stemness programs reciprocally influence telomerase activity and metabolic pathway selection. Together, these bidirectional interactions generate a self‐reinforcing feedback network that buffers GBM against single‐modality therapeutic interventions.

By anchoring GSC heterogeneity to TMMs, the Triadic Nexus further provides a mechanistically grounded taxonomy of tumor states. Distinct TMM‐defined GSC subtypes exhibit characteristic metabolic dependencies, immune phenotypes, and therapeutic vulnerabilities, offering a rational basis for precision intervention beyond descriptive classification.

### 6.2 Unanswered Questions and Emerging Technologies

Despite growing evidence supporting nexus‐based models, several fundamental questions remain unresolved. It is still unclear how stable TMM‐defined metabolic states are over time, and to what extent therapeutic pressure actively drives transitions between telomerase‐dominant and ALT‐like programs. The molecular triggers governing such state switching‐and whether they can be intercepted before adaptive resistance emerges‐remain open areas of investigation

Equally unresolved is how spatial context shapes the Triadic Nexus. GSCs residing in hypoxic, perivascular, or immune‐enriched niches likely experience distinct metabolic and telomeric constraints, yet bulk profiling approaches obscure this spatial heterogeneity. Emerging technologies such as single‐cell multi‐omics, spatial transcriptomics, and spatial metabolomics now offer the resolution necessary to map telomere maintenance, metabolic flux, and stemness programs within intact tumor architecture. Integrating these modalities will be essential for capturing the dynamic and spatially organized nature of adaptive resistance.

From a functional standpoint, direct measurement of metabolic flux, redox state, and telomere dynamics at single‐cell resolution remains technically challenging. Advances in live‐cell metabolic imaging, isotope tracing, and telomere‐specific reporters may provide critical tools to transform correlative observations into causal insight. Together, these technologies will determine whether the Triadic Nexus represents a stable organizing principle or a transient adaptive configuration shaped by environmental stress.

### 6.3 The Roadmap to Clinical Translation: From Bench to Bedside

Translating the Triadic Nexus into clinical impact requires a departure from traditional, single‐target therapeutic paradigms. Given the intrinsic adaptability of GBM, effective intervention will likely depend on rational combinatorial strategies that simultaneously constrain stemness, metabolic plasticity, and telomere maintenance. Such approaches must be informed by biomarker‐guided patient stratification, leveraging TMM status, metabolic signatures, and stemness‐associated programs to identify dominant dependencies in individual tumors.

Importantly, clinical translation will require adaptive trial designs capable of responding to dynamic tumor states. Static treatment assignments based solely on baseline genomic features are unlikely to capture the evolving biology of GBM under therapy. Instead, longitudinal monitoring of metabolic stress, telomere dysfunction, and transcriptional state may enable real‐time therapeutic adjustment, preventing adaptive escape before resistance becomes fixed.

Ultimately, the goal of nexus‐informed therapy is not simply to eliminate tumor cells but to collapse the adaptive landscape that allows GBM to persist. By targeting the regulatory circuitry that links genome maintenance, metabolism, and stemness, the Triadic Nexus framework provides a roadmap for moving beyond incremental gains toward durable disease control. While significant challenges remain, the integration of systems‐level biology with emerging technologies offers a realistic path toward transforming GBM treatment from palliative management to rational, precision‐guided intervention.

## Author Contributions

J.Y.S. conceptualized the manuscript, created the methodology, did the formal analysis, wrote the original draft, reviewed and edited, and supervised the manuscript. K.H.H. acquired the funding. Both authors have read and agreed to the published version of the manuscript.

## Funding

This research was supported by a grant of the Korea Health Technology R&D Project through the Korea Health Industry Development Institute (KHIDI), funded by the Ministry of Health & Welfare, Republic of Korea (grant number: KH129481). This work was supported by Bio Industrial Technology Development Program (20018578, Production standardization and development of analysis to verify the quality and characterization of organoid based regeneration medicine) funded by the Ministry of Trade, Industry and Energy (MOTIE, Korea).

## Ethics Statement

The authors have nothing to report.

## Conflicts of Interest

The authors declare no conflicts of interest.

## Data Availability

The authors have nothing to report.
